# Exploring the Effects of Teas with Different Fermentation Levels and Black Coffee on the Body via the Urine Proteome

**DOI:** 10.3390/nu18020343

**Published:** 2026-01-21

**Authors:** Yuzhen Chen, Youhe Gao

**Affiliations:** Gene Engineering Drug and Biotechnology Beijing Key Laboratory, College of Life Sciences, Beijing Normal University, Beijing 100875, China; 202321200036@mail.bnu.edu.cn

**Keywords:** urine, proteomics, green tea, oolong tea, black tea, Pu-erh tea, black coffee

## Abstract

**Background/Objectives:** Tea and coffee, two of the most widely consumed beverages worldwide, play important roles in supporting overall health. Changes in the urine proteome reflect the changes in the body influenced by beverage consumption, rather than beverage metabolites. In this study, the effects of teas with different fermentation levels and black coffee on the body were explored via urine proteomics analysis. **Methods:** Urine samples were collected from rats before and after seven consecutive days of consuming green tea, oolong tea, black tea, Pu-erh tea, or black coffee. Both before-and-after comparisons and between-group comparisons were performed, and the samples were analyzed using liquid chromatography coupled with tandem mass spectrometry. **Results:** The urine proteome reflected the changes in rats after consumption of teas or black coffee for one week. Biological processes and pathways enriched with differential proteins included fat cell differentiation, lipid metabolism, glucose metabolism, fatty acid transport, and immune response. The effects of teas with different fermentation levels and black coffee on the body exhibited a high degree of specificity. Additionally, several identified differential proteins have been reported as biomarkers for diseases such as cancer and cardiovascular diseases. This suggests that beverage consumption, including tea and black coffee, should be considered in urine biomarker research. And the use of biomarker panels may be necessary to improve accuracy. **Conclusions:** The urine proteome provides a comprehensive and systematic reflection of the effects of all components in teas and black coffee on the body and allows for the distinction of changes in the body after consumption of teas with different fermentation levels and black coffee.

## 1. Introduction

Tea is the second most consumed beverage in the world after water and contains a variety of bioactive components, including polyphenols, theaflavins, thearubigins, and caffeine, which possess antiviral, antioxidant, and anti-inflammatory properties [1]. Numerous studies have shown that tea has the beneficial effects of reducing the risk of cardiovascular disease, stroke [2], type 2 diabetes mellitus [3], hypertension [4], various cancers [5], and dementia [6]; improving mood [7]; and anti-obesity [8]. Tea can be classified based on the levels of fermentation into green tea (unfermented), oolong tea (partially fermented), black tea (completely fermented), and Pu-erh tea (drastically fermented and aged) [9]. The level of fermentation affects the content of bioactive components in tea [10].

Coffee is also one of the most widely consumed beverages worldwide. It contains more than a thousand compounds, including caffeine, chlorogenic acid, diterpenes, and trigonelline [11]. Coffee plays a positive role in reducing the mortality of cardiovascular diseases and the incidence of stroke [12], lowering the risk of cancer [13], Parkinson’s disease [14], and type 2 diabetes mellitus [15], as well as displaying anti-obesity effects [16].

Proteomics research reveals the composition and dynamics of proteins within cells or organisms by analyzing protein structure, expression, post-translational modifications, and protein interactions. Urine, not strictly regulated by homeostatic mechanisms, can accommodate and accumulate more changes, reflecting changes in all organs and systems of the body earlier and more sensitively [17]. Tea and coffee also contain unidentified components that may contribute to overall health. Since urinary proteins are basically not derived directly from these beverages, changes in the urine proteome reflect the changes in the body under the influence of beverages rather than the presence of beverage metabolites themselves. Thus, the use of the urine proteome offers a comprehensive and systematic approach to exploring the overall effects of all the components of these beverages.

Various factors, including age, genetics, gender, diet, and exercise, inevitably affect the urine proteome. Therefore, it is crucial to reduce the interference of extraneous factors in experiments. Animal models, whose genetic and environmental factors can be controlled, are suitable choices [18].

**Figure 1:**
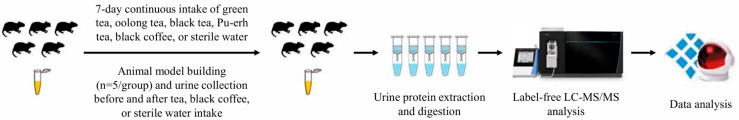
Technical route for exploring the effects of teas with different fermentation levels and black coffee on the body via the urine proteome.

Can we leverage urine’s ability to comprehensively, systematically, and sensitively reflect the body’s state to meticulously explore the effects of widely consumed tea and black coffee and seek the differences among teas with different fermentation levels and coffee? In this study, the effects of teas and coffee on rats and their differences were explored via the urine proteome, aiming to provide new clues for the study of their actions. Meanwhile, biomarkers affected by teas and coffee were explored, so as to provide references for the clinical application of urine biomarkers of diseases and related studies (Figure 1).

## 2. Materials and Methods

### 2.1. Urine Collection

Thirty healthy male Sprague Dawley (SD) rats (200 ± 20 g) aged 6–7 weeks were purchased from Beijing Vital River Laboratory Animal Technology Co., Ltd., Beijing, China. All rats were kept under standard conditions [room temperature (22 ± 1) °C, humidity 65–70%]. Animal experiments were reviewed and approved by the Ethics Committee of the College of Life Sciences, Beijing Normal University (No. CLS-AWEC-B-2022-003).

The thirty rats were randomly assigned to six groups (five rats per group). After a 3-day acclimation period under standard conditions, the rats were uniformly placed in metabolic cages to collect urine samples for 12 h. The experimental groups consumed commercial green tea, oolong tea, black tea, Pu-erh tea, or black coffee, while the control group consumed sterile water. Commercial ready-to-drink green tea, oolong tea, and black tea (Oriental Leaf, Nongfu Spring Co., Ltd., Hangzhou, Zhejiang, China) were used directly. The Pu-erh tea (Deepure Pu’er Tea Extract, Yunnan Tasly Deepure Biological Tea Group Co., Ltd., Pu’er, Yunnan, China) was prepared by dissolving 96.5 mg of tea powder in 500 mL of sterile water. The black coffee [Zhihu Zhiwu Cold Brew Coffee, Zhizhe Sihai (Beijing) Technology Co., Ltd., Beijing, China] was prepared by diluting 33 mL of coffee concentrate in 547 mL of sterile water. All the beverages and sterile water were placed in 250 mL drinking bottles, and the rats were allowed to consume food and drink ad libitum. The teas, coffee, and sterile water were replaced with new ones every day. After 7 days of treatment, rats were uniformly placed in metabolic cages to collect urine samples for 12 h. The urine samples were stored at −80 °C.

### 2.2. Urine Sample Preparation for Label-Free Analysis

The collected urine samples were centrifuged at 12,000× *g* for 40 min at 4 °C. The supernatant was transferred to a new centrifuge tube. Pre-cooled anhydrous ethanol, three times the volume of the supernatant, was added, mixed thoroughly, and then precipitated overnight at −20 °C. Following centrifugation at 12,000× *g* for 30 min at 4 °C, the supernatant was discarded. The protein precipitate was then suspended in an appropriate lysis buffer (8 mol/L urea, 2 mol/L thiourea, 25 mmol/L dithiothreitol, and 50 mmol/L Tris) and centrifuged again at 12,000× *g* for 30 min at 4 °C. The supernatant was transferred to the new centrifuge tube to obtain urine protein extract. Protein concentration was quantified using the Bradford kit assay (Applygen, Beijing, China).

Using filter-aided sample preparation (FASP) method, a total of 100 μg of protein was added to a 1.5 mL centrifuge tube. A 25 mmol/L NH_4_HCO_3_ solution was added to make a total volume of 200 μL. A 20 mmol/L dithiothreitol solution (DTT, Sigma, St. Louis, MO, USA) was added, vortexed, and mixed well. The sample was heated in a metal bath at 97 °C for 10 min. After cooling to room temperature, 50 mmol/L iodoacetamide solution (IAA, Sigma, St. Louis, MO, USA) was added, vortexed, mixed well, and reacted at room temperature without light for 40 min. A volume of 200 μL of UA solution (8 mol/L urea and 0.1 mol/L Tris-HCl, pH 8.5) was added to a 10 kD ultrafiltration tube (Pall, Port Washington, NY, USA) and centrifuged twice at 14,000× *g* for 5 min at 18 °C. The treated protein sample was added and centrifuged at 14,000× *g* for 40 min at 18 °C. A volume of 200 μL of UA solution was then added and centrifuged at 14,000× *g* for 40 min at 18 °C, repeated once. A 25 mmol/L NH_4_HCO_3_ solution was added and centrifuged at 14,000× *g* for 40 min at 18 °C, repeated once. The samples were digested overnight at 37 °C with trypsin (Trypsin Gold, Promega, Madison, WI, USA) at an enzyme-to-protein ratio of 1:50. The digested peptides were eluted from ultrafiltration membranes, desalted with HLB columns (Waters, Milford, MA, USA), dried in a vacuum desiccator, and stored at −80 °C.

### 2.3. Liquid Chromatography Coupled with Tandem Mass Spectrometry Analysis

The digested peptides were dissolved in 0.1% formic acid, and the peptide concentration was quantified using a BCA kit. The peptides were then diluted to a final concentration of 0.5 μg/μL. A mixed peptide sample was prepared by combining 3.3 μL from each sample, and separation was performed using a high-pH reversed-phase peptide fractionation kit (Thermo Fisher Scientific, Rockford, IL, USA). Ten fractions were collected by centrifugation, dried in a vacuum desiccator, and redissolved in 0.1% formic acid. The indexed retention time (iRT) reagent (Biognosis, Schlieren, Switzerland) was then added at a ratio of 1:10 (iRT-to-sample).

The ten fractions obtained from the high-pH reversed-phase peptide fractionation kit were analyzed by mass spectrometry in data-dependent acquisition (DDA) mode to generate a spectral library. A total of 1 μg of the peptide from each sample was separated using the EASY-nLC 1200 system (Thermo Fisher Scientific, Waltham, MA, USA). The sample was loaded onto a reversed-phase C18 trap column (75 μm × 2 cm, 3 μm) at a flow rate of 0.3 μL/min and separated with a reversed-phase analytical column (50 µm × 15 cm, 2 µm). A 90 min gradient elution step was applied using mobile phase A (0.1% formic acid) and mobile phase B (0.1% formic acid in 80% acetonitrile).

Analysis was performed using an Orbitrap Fusion Lumos Tribrid mass spectrometer (Thermo Fisher Scientific, Waltham, MA, USA). The spray voltage was set to 2.25 kV. A full MS scan was acquired within a 350–1550 *m*/*z* range with a resolution of 120,000. The MS/MS scan was acquired in Orbitrap mode at a resolution of 30,000. The HCD collision energy was set to 30%. The top 20 precursors were selected and subjected to a 30 s dynamic exclusion period. The DDA results were then imported into Proteome Discoverer software (version 2.1, Thermo Fisher Scientific) for searching against the *Rattus norvegicus* database using SEQUEST HT. The search results were used to establish the data-independent acquisition (DIA) method. The width and number of windows were calculated based on the *m*/*z* distribution density.

Individual samples were analyzed using the DIA mode, with each sample run in triplicate. The LC settings for DIA mode were identical to those used in DDA mode. The spray voltage was set to 2.3 kV. A full MS scan was acquired within a 350–1500 *m*/*z* range at a resolution of 60,000. The MS/MS scan was acquired in Orbitrap mode in the range of 200–2000 *m*/*z* at a resolution of 30,000. The HCD collision energy was set to 32%. After every 9–10 runs, a single DIA analysis of the pooled peptides was performed to control the quality of the whole analytical process.

### 2.4. Database Searching and Data Processing

The results were then imported into Spectronaut Pulsar (version 19, Biognosys AG, Schlieren, Switzerland) software for analysis and processing. The peptide intensity was calculated by summing the peak areas of the respective fragment ions for MS^2^, while protein intensity was calculated by summing the peptide intensities. Local regression normalization was performed. All results were filtered with a *Q* value < 0.01 (corresponding to a false discovery rate [FDR] < 1%), and only proteins containing at least two unique peptides were retained.

### 2.5. Data Analysis

In this study, both before-and-after and between-group comparisons were performed. Missing values were replaced with zeros, a practice that does not indicate an absolute zero abundance of the non-detected proteins but rather serves as a data processing strategy for subsequent statistical analysis. To minimize the effects of individual differences, differential proteins were screened by comparing urinary proteins before and after the rats consumed teas or coffee, using the criteria of fold change (FC) ≥ 1.5 or ≤0.67 and *p* < 0.05 by two-tailed paired *t*-test analysis. To avoid the effects of short-term growth and development of rats, urinary proteins were compared between the experimental and control groups using the criteria of FC ≥ 1.5 or ≤0.67 and *p* < 0.05 by two-tailed unpaired *t*-test analysis.

Hierarchical cluster analysis (HCA) and principal component analysis (PCA) were performed on differential proteins across each group comparison using the SRplot web server (http://www.bioinformatics.com.cn/, accessed on 9 January 2026). Functional enrichment analysis of differential proteins was performed using the UniProt database (https://www.uniprot.org/, accessed on 14 June 2025) and the Database for Annotation, Visualization, and Integrated Discovery (DAVID) (https://davidbioinformatics.nih.gov/, accessed on 31 May 2025). Functional analysis was further supported by searching the PubMed database (https://pubmed.ncbi.nlm.nih.gov/, accessed on 1 July 2025) for relevant studies in the literature.

## 3. Results

In this study, rats showed no obvious preference for any of the teas with different fermentation levels or coffee. Sixty samples from both the experimental and control groups were analyzed by liquid chromatography coupled with tandem mass spectrometry. A total of 1647 proteins were identified based on the criteria that each protein contained at least two unique peptides and a false discovery rate (FDR) < 1% at the protein level (Table S1).

To assess the possibility of random generation of the identified differential proteins, randomized grouping tests were performed for each group comparison on the total proteins. The ten samples involved in each group comparison (either before and after beverage consumption or between the experimental and control groups) were randomly divided into two new groups, resulting in a total of 126 combinations per group comparison. These combinations were then screened for differences based on the same criteria (FC ≥ 1.5 or ≤0.67, *p* < 0.05). The average number of differential proteins across all randomized combinations of each group comparison was calculated, and we further compared this value with the number of differential proteins identified in the original grouping to assess the possibility of the identified differential proteins being generated randomly in each group comparison. A summary of the randomized grouping test results is presented in Table 1, with detailed data listed in Table S2 (before-and-after comparisons) and Table S3 (between-group comparisons).

### 3.1. Comparative Analysis Before and After Consumption of Teas and Coffee

#### 3.1.1. Comparison Before and After Green Tea Consumption

**Figure 2:**
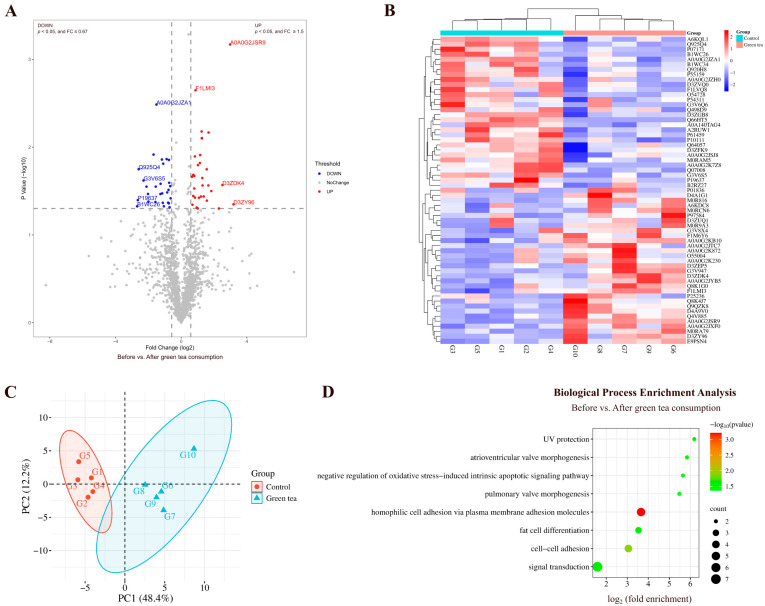
Comparative analysis before and after green tea consumption. (**A**) Volcano plot showing differential proteins: blue dots (*p* < 0.05, FC ≤ 0.67), red dots (*p* < 0.05, FC ≥ 1.5), and gray dots (non-significant). Proteins with presence–absence changes (FC = 0 or FC → ∞) were excluded from this plot due to unfeasible log_2_ transformation, and those with *p* < 0.05 are discussed in the main text. (**B**) HCA of differential proteins distinguished the samples of rats before and after green tea consumption. (**C**) PCA of differential proteins distinguished the samples of rats before and after green tea consumption. (**D**) Biological process enrichment analysis (*p* < 0.05).

By comparing the urinary proteins of rats before and after green tea consumption, a total of 61 differential proteins were identified (Figure 2A; detailed data in Table S4). Both HCA and PCA distinguished the samples of rats before and after green tea consumption (Figure 2B,C).

##### Biological Pathways Enriched with Differential Proteins in Green Tea Group

Biological process enrichment analysis of the differential proteins was performed using the DAVID database (Figure 2D; detailed data in Table S5). These differential proteins are mainly involved in biological processes including homophilic cell adhesion via plasma membrane adhesion molecules, negative regulation of oxidative stress-induced intrinsic apoptotic signaling pathway, fat cell differentiation, and pulmonary valve morphogenesis.

##### Differential Proteins Identified in Green Tea Group

Among the identified differential proteins, CCN family member 1 and Cadherin 26 exhibited changes from presence to absence (detected in at least 80% of the samples before green tea consumption but not in those after consumption). CCN family member 1 has been reported as an early marker for infarct size and left ventricular dysfunction in patients with ST-segment elevation myocardial infarction [19]. Cadherin 26 participates in calcium-dependent cell–cell adhesion via plasma membrane cell adhesion molecules, CD4-positive alpha-beta T-cell activation, and cell migration.

The 45 kDa calcium-binding protein, with the smallest *p*-value and the second-highest FC value, participates in fat cell differentiation. Studies have shown that SDF4 expression is elevated in various cancer cell types, especially those with high proliferative and metastatic potential. Serum SDF4 levels have been proposed as a diagnostic biomarker for malignancies including gastric cancer [20].

N-acetylneuraminate synthase (the third-smallest FC value) participates in carbohydrate biosynthetic process, CMP-N-acetylneuraminate biosynthetic process, glycosylation, and N-acetylneuraminate biosynthetic process.

Tissue-type plasminogen activator (the fourth-smallest FC value) participates in the regulation of synaptic plasticity, glutamatergic synaptic transmission, trans-synaptic signaling by BDNF, and responses to fatty acid.

#### 3.1.2. Comparison Before and After Oolong Tea Consumption

**Figure 3:**
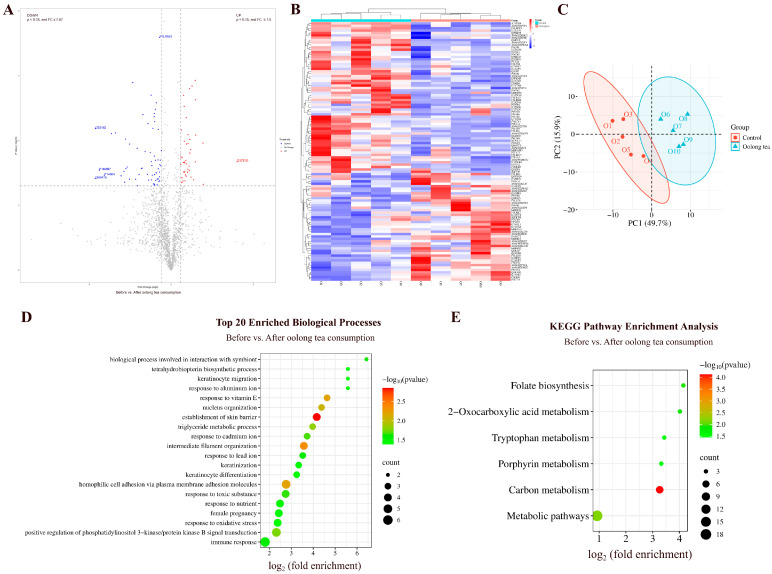
Comparative analysis before and after oolong tea consumption. (**A**) Volcano plot showing differential proteins (classification criteria consistent with Figure 2A). (**B**) HCA of differential proteins distinguished the samples of rats before and after oolong tea consumption. (**C**) PCA of differential proteins distinguished the samples of rats before and after oolong tea consumption. (**D**) Biological process enrichment analysis (top 20; *p* < 0.05). (**E**) KEGG pathway enrichment analysis (*p* < 0.05).

By comparing the urinary proteins of rats before and after oolong tea consumption, a total of 108 differential proteins were identified (Figure 3A; detailed data in Table S4). Both HCA and PCA distinguished the samples of rats before and after oolong tea consumption (Figure 3B,C).

##### Biological Pathways Enriched with Differential Proteins in Oolong Tea Group

These differential proteins are mainly involved in biological processes including establishment of skin barrier, triglyceride metabolism, positive regulation of phosphatidylinositol 3-kinase/protein kinase B signal transduction, and response to oxidative stress (Figure 3D; detailed data in Table S5). Oolong tea polyphenols have been shown to ameliorate circadian rhythm disorders by regulating the circadian rhythm oscillations of both intestinal flora and the transcription of circadian clock genes. The PI3K/Akt signaling pathway was one of the Kyoto Encyclopedia of Genes and Genomes (KEGG) pathways that was enriched with the most differentially expressed genes after oolong tea polyphenol intervention [21]. KEGG enrichment analysis revealed significant enrichment in pathways including carbon metabolism, folate biosynthesis, 2-Oxocarboxylic acid metabolism, and tryptophan metabolism (Figure 3E; detailed data in Table S6). Studies have shown that high consumption of oolong tea during pregnancy is associated with lower serum folate levels [22].

##### Differential Proteins Identified in Oolong Tea Group

CCN family member 1, with the second-smallest FC value, was also identified in the before-and-after comparison of the oolong tea group.

Catalase, which exhibited a change from presence to absence, participates in cellular detoxification of hydrogen peroxide, response to hydrogen peroxide, cholesterol metabolism, response to fatty acids, response to oxidative stress, and triglyceride metabolism. Studies have shown that unfermented oolong tea kombucha significantly increased the mRNA levels of catalase in HEK-293 cells [23].

Protocadherin-8, with the largest FC value, has been reported as a prognostic biomarker for thyroid cancer [24].

#### 3.1.3. Comparison Before and After Black Tea Consumption

**Figure 4:**
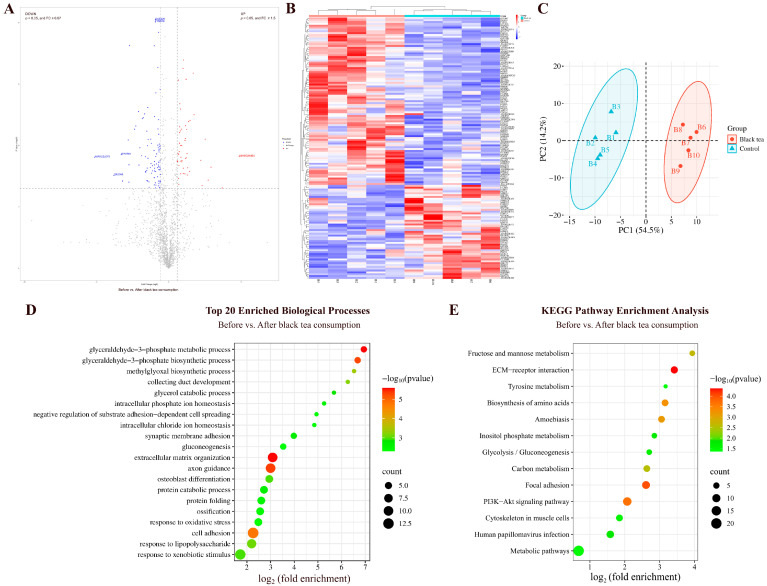
Comparative analysis before and after black tea consumption. (**A**) Volcano plot showing differential proteins (classification criteria consistent with Figure 2A). (**B**) HCA of differential proteins distinguished the samples of rats before and after black tea consumption. (**C**) PCA of differential proteins distinguished the samples of rats before and after black tea consumption. (**D**) Biological process enrichment analysis (top 20; *p* < 0.05). (**E**) KEGG pathway enrichment analysis (*p* < 0.05).

By comparing the urinary proteins from rats before and after black tea consumption, a total of 142 differential proteins were identified (Figure 4A; detailed data in Table S4). Both HCA and PCA distinguished the samples of rats before and after black tea consumption (Figure 4B,C).

##### Biological Pathways Enriched with Differential Proteins in Black Tea Group

These differential proteins are mainly involved in biological processes including glyceraldehyde-3-phosphate metabolism and biosynthesis, axon guidance, response to lipopolysaccharide, glycerol catabolism, response to oxidative stress, gluconeogenesis, canonical glycolysis, lipid metabolism, glucose metabolism, and fatty acid transport (Figure 4D; detailed data in Table S5). KEGG pathways including the PI3K-Akt signaling pathway, fructose and mannose metabolism, carbon metabolism, inositol phosphate metabolism, glycolysis/gluconeogenesis, and tyrosine metabolism were enriched (Figure 4E; detailed data in Table S6). Black tea extract has been shown to inhibit growth and induce apoptosis in HepG2 cells via the PI3K-Akt signaling pathway [25].

##### Differential Proteins Identified in Black Tea Group

Among the identified differential proteins, cytoplasmic FMR1-interacting protein exhibited a change from absence to presence (detected in at least 80% of the samples after black tea consumption but not in those before consumption). Cytoplasmic FMR1-interacting protein has been reported as a potential biomarker for diagnosing nasopharyngeal carcinoma, monitoring disease progression, and guiding therapeutic regimen selection [26].

Acid phosphatase (the second-largest FC value) participates in adenosine metabolism, nucleotide metabolism, and thiamine metabolism.

Spectrin beta chain (the smallest FC value) participates in central nervous system development, central nervous system formation, and positive regulation of interleukin-2 production. It has been reported as a biomarker for early diagnosis and precision treatment of renal clear cell carcinoma [27].

Fibulin-1 exhibited the smallest *p*-value. Studies have shown that Fibulin-1 is closely associated with the extent of target organ damage in patients at high risk of cardiovascular disease and may serve as a biomarker for risk stratification in these patients [28].

#### 3.1.4. Comparison Before and After Pu-erh Tea Consumption

**Figure 5:**
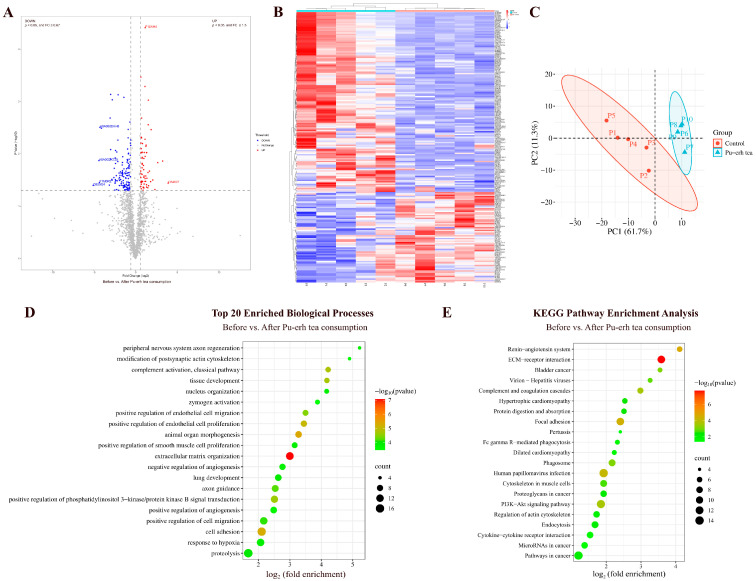
Comparative analysis before and after Pu-erh tea consumption. (**A**) Volcano plot showing differential proteins (classification criteria consistent with Figure 2A). (**B**) HCA of differential proteins distinguished the samples of rats before and after Pu-erh tea consumption. (**C**) PCA of differential proteins distinguished the samples of rats before and after Pu-erh tea consumption. (**D**) Biological process enrichment analysis (top 20; *p* < 0.05). (**E**) KEGG pathway enrichment analysis (*p* < 0.05).

By comparing the urinary proteins of rats before and after Pu-erh tea consumption, a total of 200 differential proteins were identified (Figure 5A; detailed data in Table S4). Both HCA and PCA distinguished the samples of rats before and after Pu-erh tea consumption (Figure 5B,C).

##### Biological Pathways Enriched with Differential Proteins in Pu-erh Tea Group

These differential proteins are mainly involved in biological processes including positive regulation of phosphatidylinositol 3-kinase/protein kinase B signal transduction, axon guidance, response to hypoxia, angiogenesis, response to lipopolysaccharide, immune response, and coronary vein morphogenesis (Figure 5D; detailed data in Table S5).

KEGG pathways including ECM–receptor interaction, renin–angiotensin system, focal adhesion, human papillomavirus infection, PI3K-Akt signaling pathway, pathways in cancer, hypertrophic cardiomyopathy, and dilated cardiomyopathy were enriched (Figure 5E; detailed data in Table S6). Theabrownin, a bioactive component of Pu-erh tea, has been reported to regulate glycolipid metabolism through the IRS-1/PI3K/Akt signaling pathway [29].

##### Differential Proteins Identified in Pu-erh Tea Group

Among the identified differential proteins, eight proteins—charged multivesicular body protein 2B, SPARC-like protein 1, syntaxin-7, shisa family member 7, CMRF35-like molecule 1, nebulin, proteasome activator complex subunit 1, and latent transforming growth factor beta binding protein 2—showed changes from presence to absence.

Charged multivesicular body protein 2B participates in autophagy and cognition. Syntaxin-7 participates in positive regulation of receptor localization to synapse and positive regulation of T-cell-mediated cytotoxicity. Shisa family member 7 participates in gamma-aminobutyric acid receptor clustering, gamma-aminobutyric acid signaling pathway, memory, positive regulation of long-term synaptic potentiation, and regulation of AMPA glutamate receptor clustering. CMRF35-like molecule 1 participates in the immune system process. Nebulin participates in the assembly of cardiac muscle thin filaments. Proteasome activator complex subunit 1 has been reported as an independent prognostic biomarker for soft-tissue smooth muscle sarcoma [30]. Latent transforming growth factor beta binding protein 2 has been reported as a diagnostic biomarker and potential therapeutic target for pancreatic cancer [31].

#### 3.1.5. Comparison Before and After Black Coffee Consumption

**Figure 6:**
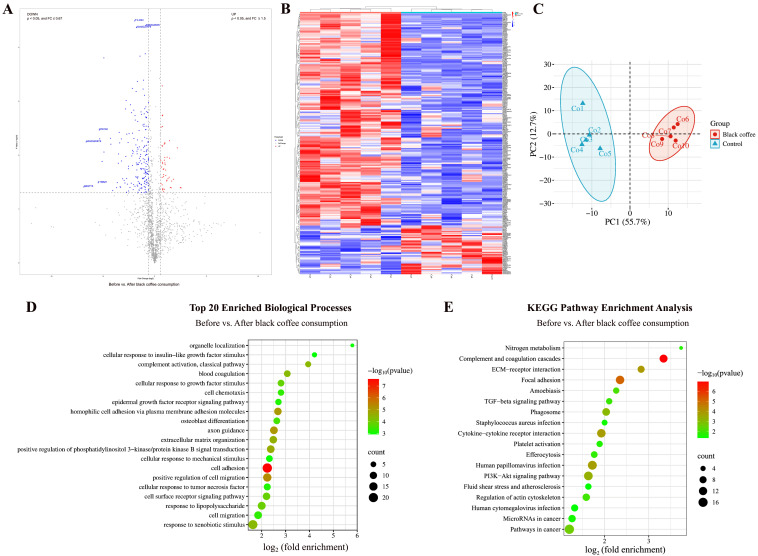
Comparative analysis before and after black coffee consumption. (**A**) Volcano plot showing differential proteins (classification criteria consistent with Figure 2A). (**B**) HCA of differential proteins distinguished the samples of rats before and after black coffee consumption. (**C**) PCA of differential proteins distinguished the samples of rats before and after black coffee consumption. (**D**) Biological process enrichment analysis (top 20; *p* < 0.05). (**E**) KEGG pathway enrichment analysis (*p* < 0.05).

By comparing the urinary proteins of rats before and after coffee consumption, a total of 246 differential proteins were identified (Figure 6A; detailed data in Table S4). Both HCA and PCA distinguished the samples of rats before and after coffee consumption (Figure 6B,C).

##### Biological Pathways Enriched with Differential Proteins in Black Coffee Group

These differential proteins are mainly involved in biological processes including axon guidance, positive regulation of phosphatidylinositol 3-kinase/protein kinase B signal transduction, response to lipopolysaccharide, ephrin receptor signaling pathway, complement activation, heart development, negative regulation of apoptotic process, and immune response (Figure 6D; detailed data in Table S5). KEGG pathways including complement and coagulation cascades, focal adhesion, PI3K-Akt signaling pathway, human papillomavirus infection, nitrogen metabolism, platelet activation, microRNAs in cancer, fluid shear stress and atherosclerosis, and pathways in cancer were enriched (Figure 6E; detailed data in Table S6). Coffee consumption has been reported as one of the risk factors for human papillomavirus infection [32]. Additionally, caffeine activates the PI3K/Akt pathway and inhibits apoptosis in the SH-SY5Y cell model of Parkinson’s disease [33].

##### Differential Proteins Identified in Black Coffee Group

Among the identified differential proteins, ten proteins—tetraspanin, RAB5B, shisa family member 7, latent transforming growth factor beta binding protein 2, tumor necrosis factor receptor superfamily member 4, choline transporter-like protein 2, glycoprotein hormone alpha-2, protocadherin alpha-4, CCN family member 1, and tissue-type plasminogen activator—showed changes from presence to absence.

Tetraspanin participates in the negative regulation of blood coagulation and regulation of gene expression. It has been reported as a diagnostic and prognostic biomarker as well as a therapeutic target in colorectal cancer [34]. RAB5B participates in antigen processing and presentation, and endocytosis. Tumor necrosis factor receptor superfamily member 4 participates in cellular defense response, inflammatory response, negative regulation of activation-induced cell death of T cells, positive regulation of B-cell proliferation, T-cell proliferation, and regulation of apoptotic process. Choline transporter-like protein 2 participates in choline transport and ethanolamine transport. Glycoprotein hormone alpha-2 participates in the adenylate cyclase-activating G protein-coupled receptor signaling pathway and cell surface receptor signaling pathway.

Matrilin 2, with the smallest *p*-value, participates in axon guidance, dendrite regeneration, glial cell migration, neuron migration, neuron projection development, and response to axon injury. It has been reported as a specific biomarker to differentiate inert from clinically aggressive pilocytic astrocytoma [35].

#### 3.1.6. Before-and-After Comparison in Control Group

By comparing the urinary proteins of rats before and after sterile water consumption, a total of 83 differential proteins were identified (Table S4). These differential proteins are mainly involved in biological processes including skeletal system morphogenesis, heart development, and cartilage development (detailed data in Table S5). Although the before-and-after comparisons minimized the effects of individual differences, they could not avoid the effects of growth and development. Therefore, to exclude the effects of short-term growth and development, between-group comparisons of urinary proteins were performed in rats from the experimental and control groups after consumption of teas, coffee, or sterile water.

### 3.2. Comparative Analysis Between Groups After Consumption of Teas and Coffee

#### 3.2.1. Comparison Between Green Tea and Control Groups

**Figure 7:**
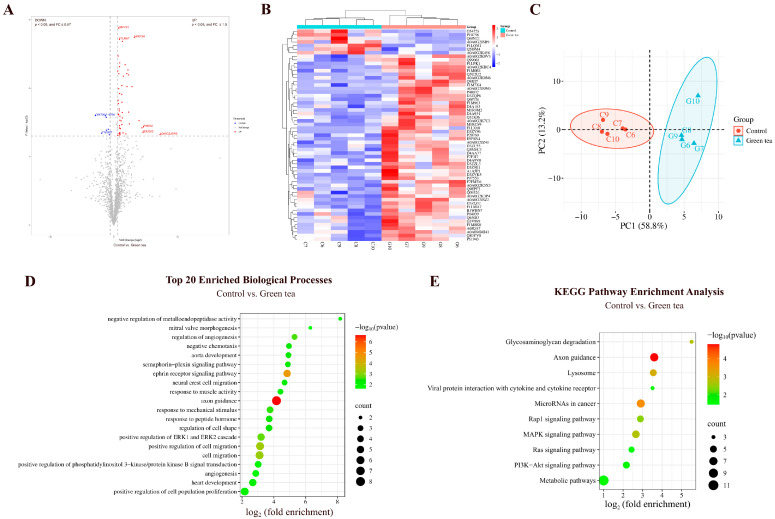
Comparative analysis between the green tea and control groups. (**A**) Volcano plot showing differential proteins (classification criteria consistent with Figure 2A). (**B**) HCA of differential proteins distinguished the samples from the green tea and control groups. (**C**) PCA of differential proteins distinguished the samples from the green tea and control groups. (**D**) Biological process enrichment analysis (top 20; *p* < 0.05). (**E**) KEGG pathway enrichment analysis (*p* < 0.05).

By comparing the urinary proteins of rats in the green tea group with those in the control group, a total of 59 differential proteins were identified (Figure 7A; detailed data in Table S7). Both HCA and PCA distinguished the samples from the green tea and control groups (Figure 7B,C).

##### Biological Pathways Enriched with Differential Proteins Between Green Tea and Control Groups

These differential proteins are mainly involved in biological processes including axon guidance, ephrin receptor signaling pathway, cell migration, positive regulation of ERK1 and ERK2 cascade, angiogenesis, aorta development, neural crest cell migration, heart development, positive regulation of phosphatidylinositol 3-kinase/protein kinase B signal transduction, and sphingosine biosynthesis (Figure 7D; detailed data in Table S8). EGCG, the major catechin in green tea, has been reported to inhibit angiogenesis through a variety of mechanisms, including induction of apoptosis and promotion of cell cycle arrest, modulation of miRNA expression profile, and inhibition of vascular endothelial growth factor (VEGF) binding to its receptor [36]. Sphingosine concentrations in plasma have been found to significantly increased 2 h after ingestion of green tea extract [37].

KEGG pathways including axon guidance, microRNAs in cancer, lysosome, MAPK signaling pathway, PI3K-Akt signaling pathway, and metabolic pathways were enriched (Figure 7E; detailed data in Table S9). Proanthocyanidins, important polyphenols in tea, have been shown to regulate miRNAs involved in cancer, and glucose and lipid homeostasis [38]. Tea polysaccharides, bioactive components of green tea, target lysosomes and induce apoptosis via a lysosomal–mitochondrial pathway-mediated caspase cascade, inhibiting proliferation of colon cancer cell line CT26 [39]. EGCG, an active ingredient in green tea, promotes the expression of protein kinase C alpha (PRKCA) and attenuates lipopolysaccharide-induced acute lung injury and inflammatory responses. This may be related to PRKCA regulating the MAPK signaling pathway and influencing the release of pro-inflammatory cytokines from macrophages [40]. Green tea extract inhibits HepG2 cell growth and induces apoptosis via the PI3K/Akt pathway [25].

##### Differential Proteins Identified Between Green Tea and Control Groups

Suppressor of tumorigenicity 14 protein homolog exhibited a change from absence to presence (detected in at least 80% of the green tea group samples but not in any of the control group samples). It has been reported to play an important role in all stages of epithelial tumorigenesis and development and holds potential as a target for anticancer therapy, as well as a diagnostic and prognostic marker [41].

Myosin light chain 12A (the second-largest FC value) is involved in the pathogenesis of inflammatory bowel disease and may serve as a new therapeutic target. Plasma Myl9 levels may also serve as a biomarker for inflammatory bowel disease [42].

Metalloproteinase inhibitor 3 (TIMP-3), with the third-largest FC value, participates in mesenchymal cell differentiation in bone development and cellular response to hypoxia and interleukin-6. TIMP-3 can be used as a surrogate marker for the response to green tea polyphenols (GTPs) and their major component epigallocatechin-3-gallate (EGCG). GTPs and EGCG inhibit prostate cancer cell migration and invasion by reactivating TIMP-3 and inhibiting MMP-2/MMP-9 activity [43].

Crk-like protein, with the fourth-largest FC value, participates in blood vessel development, B-cell apoptosis, and lipid metabolism.

Phospholipase B1 (the smallest FC value) participates in triglyceride catabolic process, retinol metabolic process, and phospholipid metabolic process. It has been identified as a potential antigen of glioblastoma, correlating with patient survival and infiltration of antigen-presenting cells, and is a potential target for glioblastoma mRNA vaccine development [44].

Lithostathine (the third-smallest FC value) participates in antimicrobial humoral immune response mediated by antimicrobial peptide and cellular response to chemokine. It has been reported to be involved in the pathophysiologic processes of Alzheimer’s disease, with a significant increase in the early stages of the disease and persistently high levels throughout the course of the disease [45].

#### 3.2.2. Comparison Between Oolong Tea and Control Groups

**Figure 8:**
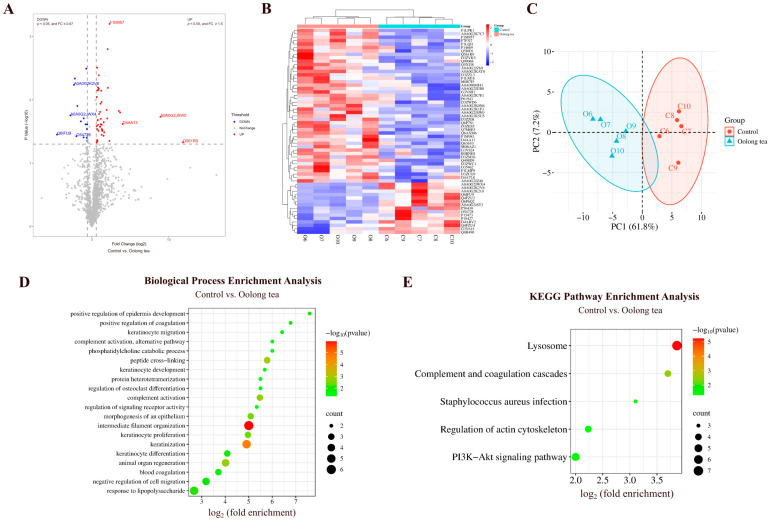
Comparative analysis between the oolong tea and control groups. (**A**) Volcano plot showing differential proteins (classification criteria consistent with Figure 2A). (**B**) HCA of differential proteins distinguished the samples from the oolong tea and control groups. (**C**) PCA of differential proteins distinguished the samples from the oolong tea and control groups. (**D**) Biological process enrichment analysis (*p* < 0.05). (**E**) KEGG pathway enrichment analysis (*p* < 0.05).

By comparing the urinary proteins of rats in the oolong tea group with those in the control group, a total of 60 differential proteins were identified (Figure 8A; detailed data in Table S7). Both HCA and PCA distinguished the samples from the oolong tea and control groups (Figure 8B,C).

##### Biological Pathways Enriched with Differential Proteins Between Oolong Tea and Control Groups

These differential proteins are mainly involved in biological processes including intermediate filament organization, keratinization, peptide cross-linking, animal organ regeneration, complement activation, morphogenesis of epithelium, response to lipopolysaccharide, positive regulation of coagulation, and phosphatidylcholine catabolism (Figure 8D; detailed data in Table S8). Studies have shown that administration of oolong tea polyphenols in mice reduces serum lipopolysaccharide levels, alleviates lipopolysaccharide-induced microglial activation, improves neuroinflammation and neuronal damage, and reduces elevated levels of the neurotoxic metabolite glutamate [46].

KEGG pathways including lysosome, complement and coagulation cascades, PI3K-Akt signaling pathway, and *Staphylococcus aureus* infection were enriched (Figure 8E; detailed data in Table S9). Studies have shown that the PI3K-Akt signaling pathway is one of the KEGG pathways that was enriched with the most differentially expressed genes following intervention with oolong tea polyphenols [21]. Additionally, water-soluble extracts from oolong tea have been shown to protect *Caenorhabditis elegans* from *Staphylococcus aureus* infection [47].

##### Differential Proteins Identified Between Oolong Tea and Control Groups

Myosin light chain 12A and phospholipase B1 were also identified as differential proteins by comparison between the oolong tea group and the control group. Selenoprotein F exhibited a change from absence to presence. *SELENOF* gene polymorphisms and SELENOF dysregulation are closely related to diseases including cancer and neurodegenerative diseases. Since SELENOF is sensitive to selenium, it may serve as a therapeutic target in the pathological processes of related diseases [48].

Endoribonuclease LACTB2 (the second-largest FC value) has been reported as a biomarker for predicting the prognosis of radiotherapy in nasopharyngeal carcinoma and as a potential therapeutic target for improving the radiosensitivity of nasopharyngeal carcinoma [49].

Type I keratin KA11, with the smallest FC value, participates in inflammatory response, establishment of skin barrier, keratinization, and morphogenesis of epithelium.

Von Willebrand factor (the smallest *p*-value) has been identified as a biomarker for diagnosing clinically significant portal hypertension (CSPH) and severe portal hypertension (SPH) in patients with cirrhosis, showing a moderate correlation with hepatic venous pressure gradient (HVPG) measurements [50]. VWF is also a biomarker for assessing the severity of valvular heart disease, evaluating treatment efficacy, and predicting patient prognosis [51].

#### 3.2.3. Comparison Between Black Tea and Control Groups

**Figure 9:**
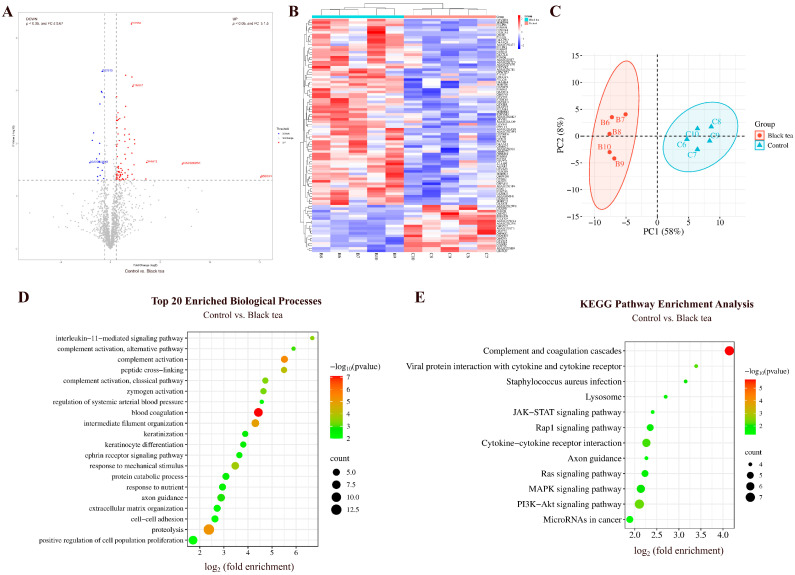
Comparative analysis between the black tea and control groups. (**A**) Volcano plot showing differential proteins (classification criteria consistent with Figure 2A). (**B**) HCA of differential proteins distinguished the samples from the black tea and control groups. (**C**) PCA of differential proteins distinguished the samples from the black tea and control groups. (**D**) Biological process enrichment analysis (top 20; *p* < 0.05). (**E**) KEGG pathway enrichment analysis (*p* < 0.05).

By comparing the urinary proteins of rats in the black tea group with those in the control group, a total of 94 differential proteins were identified (Figure 9A; detailed data in Table S7). Both HCA and PCA distinguished the samples from the black tea and control groups (Figure 9B,C).

##### Biological Pathways Enriched with Differential Proteins Between Black Tea and Control Groups

These differential proteins are mainly involved in biological processes including blood coagulation, complement activation, proteolysis, interleukin-11-mediated signaling pathway, axon guidance, and ephrin receptor signaling pathway (Figure 9D; detailed data in Table S8).

KEGG pathways including complement and coagulation cascades, PI3K-Akt signaling pathway, viral protein interaction with cytokine and cytokine receptor, MAPK signaling pathway, Ras signaling pathway, JAK-STAT signaling pathway, and microRNAs in cancer were enriched (Figure 9E; detailed data in Table S9). Studies have shown that black tea extract inhibits the proliferation of HepG2 cells and induces apoptosis via the PI3K-Akt signaling pathway [25]. Black tea can also inhibit tumor-induced thymic involution through mechanisms including preventing the downregulation of IL-7Rα in thymocytes, maintaining the phosphorylation of JAK3 and STAT5, and protecting the JAK-STAT signaling pathway [52].

##### Differential Proteins Identified Between Black Tea and Control Groups

Selenoprotein F, suppressor of tumorigenicity 14 protein homolog, Dmx-like 1, and von Willebrand factor were also identified as differential proteins by comparison between the black tea group and the control group. Selenoprotein F and suppressor of tumorigenicity 14 protein homolog exhibited changes from absence to presence.

Synaptobrevin homolog YKT6 exhibited the third-largest FC value. Studies have shown that *YKT6* can serve as an independent prognostic biomarker and a potential immunotherapy target for oral squamous cell carcinoma [53].

Keratin 2 (the smallest FC value) participates in keratinocyte activation, development, migration, proliferation, intermediate filament organization, and keratinization.

Ephrin-A1 (the smallest *p*-value) participates in angiogenesis, negative/positive regulation of MAPK cascade, aortic valve morphogenesis, and axon guidance.

Complement factor B (the second-smallest *p*-value) participates in complement activation, positive regulation of apoptotic cell clearance, and responses to bacteria, lipopolysaccharide, and nutrients.

#### 3.2.4. Comparison Between Pu-erh Tea and Control Groups

**Figure 10:**
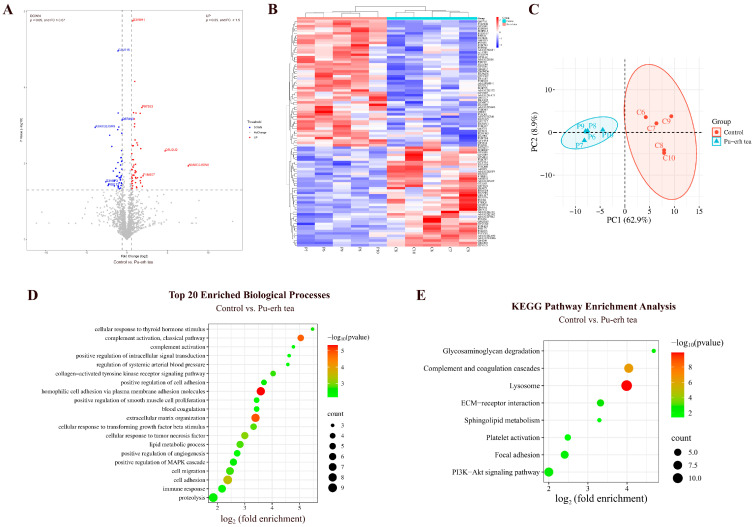
Comparative analysis between the Pu-erh tea and control groups. (**A**) Volcano plot showing differential proteins (classification criteria consistent with Figure 2A). (**B**) HCA of differential proteins distinguished the samples from the Pu-erh tea and control groups. (**C**) PCA of differential proteins distinguished the samples from the Pu-erh tea and control groups. (**D**) Biological process enrichment analysis (top 20; *p* < 0.05). (**E**) KEGG pathway enrichment analysis (*p* < 0.05).

By comparing the urinary proteins of rats in the Pu-erh tea group with those in the control group, a total of 95 differential proteins were identified (Figure 10A; detailed data in Table S7). Both HCA and PCA distinguished the samples from the Pu-erh tea and control groups (Figure 10B,C).

##### Biological Pathways Enriched with Differential Proteins Between Pu-erh Tea and Control Groups

These differential proteins are mainly involved in biological processes including homophilic cell adhesion via plasma membrane adhesion molecules, inflammatory response, complement activation, cellular response to tumor necrosis factor, lipid metabolism, negative regulation of lipid storage, regulation of systemic arterial blood pressure, glycoside catabolism, hyaluronan metabolism, and mitral valve morphogenesis (Figure 10D; detailed data in Table S8).

KEGG pathways including lysosome, complement and coagulation cascades, ECM–receptor interaction, PI3K-Akt signaling pathway, glycosaminoglycan degradation, and sphingolipid metabolism were enriched (Figure 10E; detailed data in Table S9). Studies have shown that theabrownin, a bioactive compound in black tea, regulates glycolipid metabolism via the IRS-1/PI3K/Akt signaling pathway [29]. Additionally, Pu-erh tea extract has been reported to regulate alcohol-induced metabolic disorders via pathways including sphingolipid metabolism [54].

##### Differential Proteins Identified Between Pu-erh Tea and Control Groups

Suppressor of tumorigenicity 14 protein homolog, 45 kDa calcium-binding protein, myosin light chain 12A, Crk-like protein, von Willebrand factor, ephrin-A1, and complement factor B were also identified as differential proteins by comparison between the Pu-erh tea group and the control group. Suppressor of tumorigenicity 14 protein homolog exhibited a change from absence to presence. The 45 kDa calcium-binding protein had the smallest FC value. Myosin light chain 12A exhibited the second-largest FC value. Complement factor B exhibited the second-smallest *p*-value.

Cadherin, EGF LAG seven-pass G-type receptor 2 (the second-smallest FC value) participates in cerebrospinal fluid secretion, motor neuron migration, neural plate anterior/posterior regionalization, ventricular system development, and Wnt signaling pathway.

Cytochrome b5 (the third-smallest FC value) has been reported as a novel biomarker for visceral obesity intervention and a safe therapeutic target [55].

Tissue kallikrein (the smallest *p*-value) participates in positive regulation of acute inflammatory response, positive regulation of apoptotic process, and regulation of systemic arterial blood pressure.

Acid ceramidase (the third-smallest *p*-value) participates in cellular response to tumor necrosis factor, ceramide biosynthetic process, ceramide catabolic process, fatty acid metabolic process, regulation of programmed necrotic cell death, regulation of steroid biosynthetic process, and sphingosine biosynthetic process.

Cadherin-13 has been reported to interfere with the differentiation potential of adipocytes, serving as a marker of adipose tissue plasticity and reflecting the health status of adipose tissue [56].

#### 3.2.5. Comparison Between Black Coffee and Control Groups

**Figure 11:**
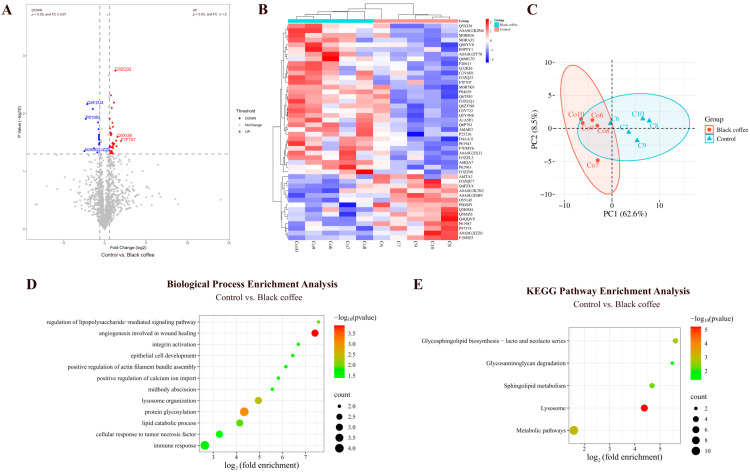
Comparative analysis between the black coffee and control groups. (**A**) Volcano plot showing differential proteins (classification criteria consistent with Figure 2A). (**B**) HCA of differential proteins distinguished the samples from the black coffee and control groups. (**C**) PCA of differential proteins distinguished the samples from the black coffee and control groups. (**D**) Biological process enrichment analysis (*p* < 0.05). (**E**) KEGG pathway enrichment analysis (*p* < 0.05).

By comparing the urinary proteins of rats in the coffee group with those in the control group, a total of 46 differential proteins were identified (Figure 11A; detailed data in Table S7). Both HCA and PCA distinguished the samples from the black coffee and control groups (Figure 11B,C).

##### Biological Pathways Enriched with Differential Proteins Between Black Coffee and Control Groups

These differential proteins are mainly involved in biological processes including angiogenesis involved in wound healing, protein glycosylation, lysosome organization, regulation of lipopolysaccharide-mediated signaling pathway, lipid catabolic process, immune response, and positive regulation of calcium ion import (Figure 11D; detailed data in Table S8). KEGG pathways including lysosome, metabolic pathways, glycosphingolipid biosynthesis (lacto and neolacto series), sphingolipid metabolism, and glycosaminoglycan degradation were enriched (Figure 11E; detailed data in Table S9).

##### Differential Proteins Identified Between Black Coffee and Control Groups

Adhesion G protein-coupled receptor F5, with the smallest FC value, participates in energy reserve metabolism, fat cell differentiation, glucose homeostasis, macrophage activation, negative regulation of macrophage activation, and phospholipid biosynthesis. ADGRF5 has been reported as a prognostic biomarker for renal clear cell carcinoma [57].

Further, 14-3-3 protein gamma (the second-smallest FC value) participates in cellular response to insulin stimulus, negative regulation of TORC1 signaling, regulation of synaptic plasticity, and cellular response to glucose starvation. YWHAG has been reported as a diagnostic biomarker for cognitive impairment in patients with Parkinson’s disease [58].

Enhancer of mRNA-decapping protein 4 (the largest FC value) participates in the deadenylation-independent decapping of nuclear-transcribed mRNA and nervous system development.

Lymphocyte cytosolic protein 1 (the second-largest FC value) participates in protein kinase A signaling and T-cell activation involved in immune response. Studies have shown that LCP1 may serve as a potential therapeutic target for obesity-related metabolic disorders [59].

## 4. Discussion

The results of this study indicate that the urine proteome can reflect changes in rats after seven consecutive days of consuming teas with different fermentation levels and black coffee. Biological processes and pathways enriched with differential proteins included fat cell differentiation, lipid metabolism, glucose metabolism, fatty acid transport, and immune response. The urinary proteins of rats in the control group (consuming sterile water) before and after 7 days were also compared in the experiment. The identified differential proteins are mainly involved in biological processes including skeletal system morphogenesis, heart development, and cartilage development. These results indicate that the urine proteome can reflect short-term growth and development changes in rats, consistent with the previous results of our laboratory [60]. Therefore, to minimize the influence of various factors, before-and-after and between-group comparisons were performed for analysis. By minimizing the influence of individual differences through before-and-after comparisons and avoiding the effects of short-term growth and development in rats through between-group comparisons, the effects of teas and coffee and their differences were meticulously explored.

**Figure 12:**
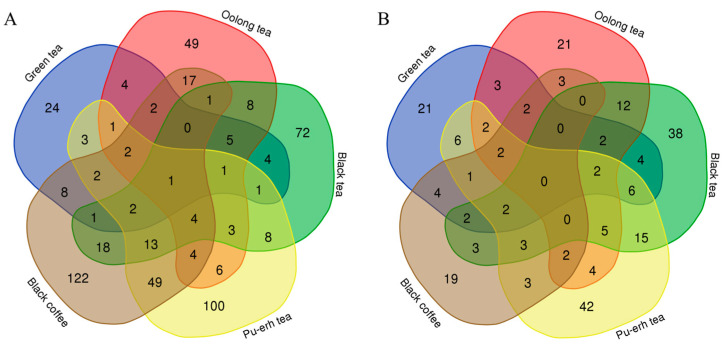
Venn diagrams of differential proteins across the five experimental groups. Numbers indicate the count of differential proteins. (**A**) Before-and-after comparisons. (**B**) Between-group comparisons.

**Figure 13:**
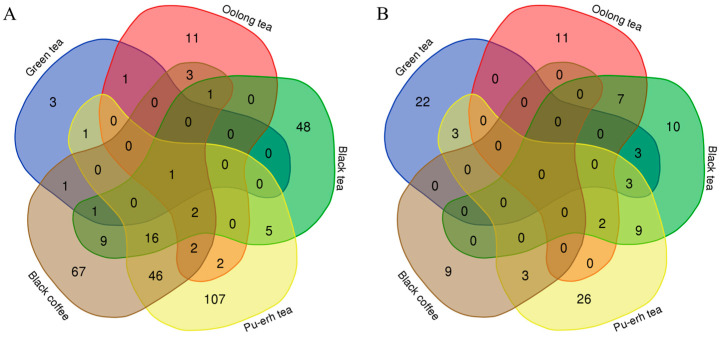
Venn diagrams of biological processes enriched with differential proteins across the five experimental groups. Numbers indicate the count of biological processes. (**A**) Before-and-after comparisons. (**B**) Between-group comparisons.

**Figure 14:**
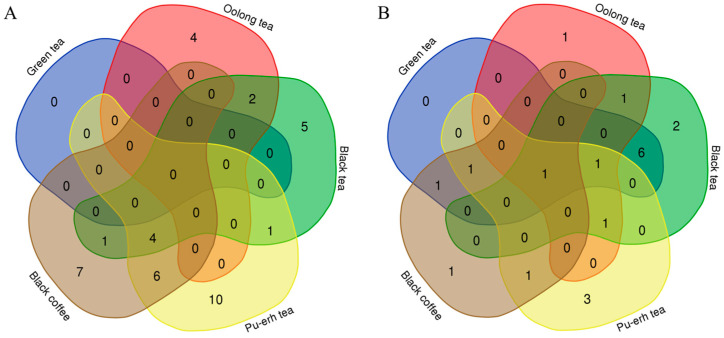
Venn diagrams of KEGG pathways enriched with differential proteins across the five experimental groups. Numbers indicate the count of KEGG pathways. (**A**) Before-and-after comparisons. (**B**) Between-group comparisons.

Venn diagrams were used to show the overlap of differential proteins identified from the before-and-after and between-group comparisons in the four tea groups and the coffee group (Figure 12). The results showed that each group shared few common differential proteins, while many were unique to each group. Similarly, Venn diagrams of biological processes (Figure 13) and KEGG pathways (Figure 14) enriched with differential proteins across the five experimental groups revealed few shared elements and many unique ones among the groups. These findings suggest that analysis of the urine proteome can distinguish the effects of teas with different fermentation levels and coffee and that these effects are highly specific to the types of beverage consumed.

Additionally, this study found that multiple identified differential proteins have been reported as biomarkers for diseases including cancer and cardiovascular diseases, particularly those proteins that exhibit significant changes under the influence of teas and coffee. This provides references for research on urine biomarkers of diseases and related studies, suggesting that future research and clinical applications of urine biomarkers of diseases should consider the potential influence of beverage consumption, including tea and coffee. During clinical urine sample collection, it is necessary to consider whether beverage restrictions are required. Additionally, relying on a single biomarker may lead to false positives, so it may be necessary to use biomarker panels to improve accuracy.

## 5. Conclusions

In conclusion, the urine proteome comprehensively and systematically reflects changes in rats after seven consecutive days of consuming four types of teas or black coffee and allows for the distinction of the changes in the body after consumption of teas with different fermentation levels and coffee.

## Figures and Tables

**Table 1:** Results of randomized grouping tests.

Groups	Number of Differential Proteins	Number of Randomly Generated Differential Proteins	Percentage of Reliable Differential Proteins
Before vs. after green tea consumption	61	39.84	34.69%
Before vs. after oolong tea consumption	108	44.60	58.70%
Before vs. after black tea consumption	142	40.44	71.52%
Before vs. after Pu-erh tea consumption	200	41.91	79.05%
Before vs. after black coffee consumption	246	44.96	81.72%
Before vs. after sterile water consumption	83	40.90	50.72%
Control vs. green tea	59	37.69	36.12%
Control vs. oolong tea	60	40.48	32.53%
Control vs. black tea	94	41.39	55.97%
Control vs. Pu-erh tea	95	34.63	63.55%
Control vs. black coffee	46	37.21	19.11%

## Data Availability

The mass spectrometry proteomics data have been deposited to the ProteomeXchange Consortium (http://proteomecentral.proteomexchange.org (accessed on 3 July 2025)) via the iProX partner repository with dataset identifier PXD065728).

## Supplementary Materials

The following supporting information can be downloaded at: https://www.mdpi.com/article/10.3390/nu18020343/s1, Table S1: Total proteins identified in the experiment, Table S2: Results of randomized grouping tests with before-and-after comparisons, Table S3: Results of randomized grouping tests with between-group comparisons, Table S4: Differential proteins identified by before-and-after comparisons, Table S5: Biological process enrichment analysis of differential proteins identified by before-and-after comparisons, Table S6: KEGG pathway enrichment analysis of differential proteins identified by before-and-after comparisons, Table S7: Differential proteins identified by between-group comparisons, Table S8: Biological process enrichment analysis of differential proteins identified by between-group comparisons, Table S9: KEGG pathway enrichment analysis of differential proteins identified by between-group comparisons.
